# Active commuting to and from university, obesity and metabolic syndrome among Colombian university students

**DOI:** 10.1186/s12889-018-5450-5

**Published:** 2018-04-19

**Authors:** Antonio García-Hermoso, Andrea P. Quintero, Enrique Hernández, Jorge Enrique Correa-Bautista, Mikel Izquierdo, Alejandra Tordecilla-Sanders, Daniel Prieto-Benavides, Carolina Sandoval-Cuellar, Katherine González-Ruíz, Emilio Villa-González, Robinson Ramírez-Vélez

**Affiliations:** 1 Laboratorio de Ciencias de la Actividad Física, el Deporte y la Salud, Facultad de Ciencias Médicas, Universidad de Santiago de Chile, USACH, Santiago, Chile; 2 Centro de Estudios para la Medición de la Actividad Física «CEMA». Escuela de Medicina y Ciencias de la Salud, Universidad del Rosario, Bogotá D.C, Colombia; 3 Department of Health Sciences, Public University of Navarre, CIBERFES (CB16/10/00315), Navarre, Spain; 4 Programa de Fisioterapia, Facultad de Ciencias de la Salud, Universidad de Boyacá, Facultad de Ciencias de la Salud, Boyacá, 150003 Colombia; 5 Grupo de Ejercicio Físico y Deportes, Vicerrectoría de Investigaciones, Universidad Manuela Beltrán, Bogotá D.C, Colombia; 6 PROFITH ¨PROmoting FITness and Health through Physical Activity¨ Research Group, Department of Physical Education and Sport, School of Sport Sciences, University of Granada, Granada, Spain

**Keywords:** Active transport, Physical activity, Cardiometabolic risk, Young adults

## Abstract

**Background:**

There is limited evidence concerning how active commuting (AC) is associated with health benefits in young. The aim of the study was to analyze the relationship between AC to and from campus (walking) and obesity and metabolic syndrome (MetS) in a sample of Colombian university students.

**Methods:**

A total of 784 university students (78.6% women, mean age = 20.1 ± 2.6 years old) participated in the study. The exposure variable was categorized into AC (active walker to campus) and non-AC (non/infrequent active walker to campus: car, motorcycle, or bus) to and from the university on a typical day. MetS was defined in accordance with the updated harmonized criteria of the International Diabetes Federation criteria.

**Results:**

The overall prevalence of MetS was 8.7%, and it was higher in non-AC than AC to campus. The percentage of AC was 65.3%. The commuting distances in this AC from/to university were 83.1%, 13.4% and 3.5% for < 2 km, 2-5 km and > 5 km, respectively. Multiple logistic regressions for predicting unhealthy profile showed that male walking commuters had a lower probability of having obesity [OR = 0.45 (CI 95% 0.25–0.93)], high blood pressure [OR = 0.26 (CI 95% 0.13–0.55)] and low HDL cholesterol [OR = 0.29 (CI 95% 0.14–0.59)] than did passive commuters.

**Conclusions:**

Our results suggest that in young adulthood, a key life-stage for the development of obesity and MetS, AC could be associated with and increasing of daily physical activity levels, thereby promoting better cardiometabolic health.

## Background

Metabolic syndrome (MetS) is a complex clustering of cardiovascular risk factors, such as abdominal obesity, hypertension, diabetes and dylipedemia [1], and consists of several cardiovascular risk factors that coexist and promote cardiovascular morbidity and mortality [2]. Accumulating data point towards the beneficial role of physical activity (PA) on promoting the control of individual cardiovascular risk factors, such as obesity, diabetes mellitus, and hypertension [3]. However, physical inactivity is a growing contributing cause to at least 35 unhealthy conditions, including the MetS, worldwide [4]. For example, in Colombia, it is estimated that only 51.3% of men and women aged 18–64 years achieve recommended daily levels of physical activity [5]. In this sense, one of the key changes in young adult life occurs during the transition from high school to university, and a decrease in PA levels is associated with this period [6]. University life appears to keep students physically inactive for long periods, which results in a reduction of the overall PA practice without meeting the recommended PA levels [7].

One important factor that contributes to low PA levels is an increase in the use of passive modes of transportation [8]. Walking and cycling to university may offer young people an opportunity to incorporate physical activity into their daily lives. Active commuting (AC) as a daily behavior is considered an opportunity to create a healthy habit, increasing PA levels and reducing the risk of cardiovascular disease, an improved metabolic profile and many other noted health benefits in youth [9, 10] and adult populations [11]. However, limited studies have analyzed AC in the collegiate student population and its positive benefits [12–14]. In two successive studies, Bopp et al. [12, 13] suggested that active travelers had greater cardiovascular fitness, were more flexible, and had lower systolic blood pressure and probability to be overweight compared with non-active travelers. In addition, Gordon-Larsen et al. [14] suggested that AC was positively associated with fitness in young adult men and women (18–30 years old) and was inversely associated with body mass index (BMI), central obesity, triglyceride levels, blood pressure, and insulin levels in males.

AC is an important strategy to promote PA in keeping with recommendations proposed by international bodies and the high rates of physical inactivity and sedentary lifestyles among students in Bogotá (Colombia). However, to the best of our knowledge, this study is the first to analyze the AC behavior of Latino university students. Thus, our study analyzed the relationship between AC to and from university (walking) and obesity and MetS in a sample of Colombian university students.

## Methods

### Study design and sample population

During the 2014–2017 academic years, we reviewed a cross-sectional component of the FUPRECOL (in Spanish Asociación de la Fuerza Prensil con Manifestaciones Tempranas de Riesgo Cardiovascular en Adultos Colombianos) Adults study, which investigated the association between muscular strength and cardiometabolic risk factors in a sample of Colombian university students. We recently published a complete description of the FUPRECOL Adults study design, methods, and primary outcomes for our current cohort [15]. The original sample consisted of adults (men: *n* = 706; women: *n* = 1126). From this subgroup, 784 university students (78.6% women) had valid data in AC and all components included in the cardiometabolic variables and AC survey. There were no differences in the study key characteristics (i.e., age, sex distribution, BMI, and MetS components) between the current study sample and the original FUPRECOL Adults Study sample (*n* = 1832, all *p* > 0.100). Data were collected in Tunja, a city in the Eastern Range of the Colombian Andes in a region known as the Altiplano Cundiboyacense, located 130 km northeast of Bogotá. In 2016, this city had an estimated population of 191,878 inhabitants. The city center is at an elevation of 2820 m (9,250 ft) above sea level. Tunja is an important educational center and is home to several well-known universities.

### Measurements

#### Anthropometry and body fatness

Subjects were tested while wearing light clothing (shoes were removed). Height (Seca® 274, Hamburg, Germany), body mass (Model Tanita® BC-420®, Tokyo, Japan) and waist circumference (WC, Lufkin W606 PM®, Parsippany, NJ, USA) were assessed according to international standards for anthropometric assessment [16]. The height and body mass measurements were used to calculate the BMI = body mass (kg)/height(m)^2^]. Trained researchers performed the measurements according to standardized procedures. The technical error of measurement values was less than 2% for all anthropometric variables. BF% was determined by a tetrapolar whole body impedance (Tanita Model BC-420®, Tokyo, Japan). A detailed description of the bioelectrical impedance analysis technique was presented in a previous study [17]. The corresponding intra-observer technical error (% reliability) of the measurements was 95%.

### Cardiometabolic variables

Blood pressure was obtained using an automatic monitor (Omrom® HEM 705 CP, Health-care Co, Kyoto, Japan) following a previously described protocol for the European Heart Society (on the right arm with participants in a supine position and subjects rested quietly for 10 min in a comfortable chair). The mean arterial blood pressure (MAP) was calculated as: (2 x diastolic)+systolic]/3 [18].

Blood samples (40 μL) were drawn between 07:00 and 09:00, after 10–12 h of fasting (11.2 h on average). Hematological entities, including total cholesterol (TC), high-density lipoprotein cholesterol (HDL-C), and triglycerides (TG), were measured by using an automated biochemical analyzer Cardiocheck® equipment (Mexglobal SA, Parsippany, NJ, USA). Low-density lipoprotein-cholesterol (LDL-C) was calculated using Friedewald’s Formula when triglyceride values were ≤ 400 mg/dL [19]. Glucose was measured using Accu-Chek® Aviva Plus system (Roche®, Mexglobal SA, Terrytown, NY, USA).

MetS was defined in accordance with the updated harmonized criteria of the International Diabetes Federation/National Heart, Lung and Blood Institute/American Heart Association (IDF/NHLBI/AHA-2009) [20]. Participants were considered to have MetS if they showed three or more of the following: (1) central obesity in WC (♀ ≥ 80 cm and ♂ ≥90 cm); (2) TG (≥150 g/dL); (3) low HDL-C (♀ < 50 mg/dL and ♂ < 40 mg/dL); (4) high blood pressure (systolic blood pressure ≥ 130 mmHg or diastolic blood pressure ≥ 85 mmHg); (5) high TG (≥100 mg/dL). The latest (IDF/NHLBI/AHA-2009) consensus stated that WC was measured according to Country/Specific values which, for Latin Americans, were set to be equal to South Asian parameters, specifically WC ≥90 cm for males and ≥ 80 cm for women [20].

### Modes of commuting to/from the university

The mode of commuting to and from school was measured using a questionnaire developed and validated in Spanish by the University of Granada (Spain) through the project “PACO: pedalea y anda al colegio” (http://profith.ugr.es/paco). This questionnaire has been validated in a Spanish population [21, 22]. The usual mode of commuting to and from campus was categorized into “active” commuting (active walker to campus) and non-active commuting (non/infrequent active walker to campus: car, motorcycle, or bus). Although this particular question for assessing commuting to/from university has not been formally validated, it is highly similar to other 1-item questionnaires on a students’ commuting to university that have been demonstrated to be acceptably reliable and valid in this age group [23]. For commuting distance from home to their university participants could choose any of the following options: 0 to 2 km, 2 to 5 km, and > 5 km. In our sample, the results of analysis internal consistency reveal that Colombian version of the project “PACO: pedalea y anda al colegio” scale have adequate to good values (commuting to/from university and barriers domain α Cronbach = 0.761, and total commuting distance from home to their university or vice versa domain α Cronbach = 0.995). Finally, due to a limited number of students who cycle to the university (*n* = 9), we have removed these individuals from the study population.

### Lifestyle covariates

A validated questionnaire, the “FANTASTIC” lifestyle, was used to collect comprehensive information about substance use via a personal interview with participants. Alcohol consumption (No or 1 to 10 cigarettes per day), smoking status (No or 1 to 9 times per week), and physical activity levels (5 times a week for > 30 min or ≥ 150 min per week were categorized as “physically active”) has been previously described by Ramírez-Vélez et al. [24].

The seven-day recall dietary assessment tool was used to complete the MetDiet. As suggested by Thanapoulou et al. [25], the total score was divided into two categories of Mediterranean diet quality: ≤8 points = poor diet quality and ≥ 9 points = good diet quality (optimal Mediterranean diet style). Participants who had ≥9 points were categorized as having an ideal healthy diet, whereas those with 8 points or less were classified as having a non-ideal diet. Personal, family history of CVD, and medication use was used as covariables.

### Ethics statement

The study was approved by the Research Ethics Committee at the Universidad Manuela Beltrán (UMB Code N° 01–1802-2013) in according the Helsinki Declaration Accord (World Medical Association for Human Subjects, 2000). Informed consent was obtained from each participant before being enrolled in the study.

### Statistical analysis

Anthropometric and body composition components and cardiometabolic risk factors of the study sample are presented as the mean (SD) or relative frequency, n (%). The normality of the variables was verified using histograms and Q-Q plots. Differences on cardiometabolic parameters between walking and non-walking groups were assessed using an ANOVA or X^2^ test. Finally, to examine the OR and 95% CI of having an unhealthy profile (MetS components), we used multinomial logistic regression by sex. The exposure variable was categorized into AC (active walker to campus) and non-AC (non/infrequent active walker to campus: car, motorcycle, or bus) to and from the university on a typical day. These analyses were adjusted by age, BMI (only when obesity or waist circumference were not included as dependent variable), PA, alcohol and tobacco intake, diet, and distance and were performed using SPSS version 21.0 for Windows (IBM, Armonk, New York). Statistical significance was established at *p* < 0.05.

## Results

The sample consisted of 784 university students from Tunjá, Boyacá. Characteristics of the whole sample were categorized in passive and active commuters are shown in Table 1. In the present study, the percentage of active commuters (i.e., walkers to/from the university) was 65.3%. The commuting distances in this active commuters from/to university were 83.1%, 13.4% and 3.5% for < 2 km, 2-5 km and > 5 km, respectively.

**Table 1:** Characteristics of the sample

	Total (*n* = 784)	Non/infrequent active walker to campus (*n* = 272)	Active walker to campus (*n* = 512)	*p*
Sex				
Male, n (%)	168 (21.4)	95 (34.9)	73 (14.3)	
Female, n (%)	616 (78.6)	177 (65.1)	439 (85.7)	
Age	20.0 (2.6)	20.1 (2.7)	20.0 (2.6)	0.558
Life-style				
Tobacco (1 to 10 cigarettes per day), n (%)^a^	213 (28.7)	74 (27.2)	139 (28.8)	0.939
Alcohol (1 to 9 times per week), n (%)^a^	371 (47.8)	128 (47.1)	243 (47.9)	0.927
PA levels (≥150 min per week), n (%)^a^	177 (22.7)	77 (28.3)	100 (19.7)	0.006
MetDiet (≥ 9 points), n (%)^a^	247 (31.5)	73 (27.0)	184 (36.0)	0.430
Anthropometric and body composition				
Weight	60.7 (11.7)	61.8 (12.5)	60.2 (11.3)	0.058
Height	161.4 (8.0)	163.4 (8.6)	160.3 (7.5)	< 0.001
Body mass index	23.2 (3.8)	23.1 (4.0)	23.3 (3.6)	0.398
Obese, n (%)^a^	212 (27.0)	70 (25.7)	142 (27.7)	0.547
Fat mass, %	24.9 (8.4)	22.9 (9.1)	26.0 (7.7)	< 0.001
Fat mass, kg	15.6 (7.5)	14.6 (8.2)	16.1 (7.2)	0.010
Cardiometabolic risk factors				
Waist circumference, cm	72.4 (8.0)	73.6 (9.5)	71.7 (8.0)	0.004
Increased waist circumference, n (%)^a^	104 (13.4)	37 (13.8)	67 (13.2)	0.818
Triglycerides, mg/dL	89.3 (44.7)	95.9 (50.0)	85.7 (41.3)	0.003
High triglycerides, n (%)^a^	68 (8.7)	29 (10.8)	39 (7.7)	0.143
HDL cholesterol, mg/dL	40.6 (11.5)	41.4 (11.3)	40.1 (11.6)	0.132
Low HDL cholesterol, n (%)^a^	582 (74.9)	188 (69.1)	342 (66.9)	0.601
Fasting plasma glucose, mg/dL	87.7 (9.2)	87.8 (9.7)	87.6 (8.9)	0.780
High fasting plasma glucose	77 (9.8)	28 (10.4)	49 (9.6)	0.728
Mean blood pressure, mmHg	92.9 (9.1)	92.3 (8.8)	93.2 (9.2)	0.204
High blood pressure	165 (21.2)	60 (22.0)	100 (19.6)	0.418
Metabolic syndrome, n (%)^a^	67 (8.7)	24 (9.1)	43 (8.5)	0.813

^a^Prevalence. MetDiet ≥9 points = good diet quality (optimal Mediterranean diet style)

There were no significant differences for the majority of the study variables between active and passive commuters to and from the university. Regarding lifestyle, a greater percentage of PA levels (≥150 min per week) were shown in passive compared to active commuters (28.3% vs. 19.7% respectively; *p* = 0.006). The passive commuters showed a higher height (*p* < 0.001) but lower % and kg of fat mass (*p* < 0.001 and* p* = 0.010, respectively) than active commuters. With respect to cardiometabolic risk factors, there were only significant differences in waist circumference (*p* = 0.004) and triglycerides (*p* = 0.003) with higher values in the passive commuters. The overall prevalence of MetS was 8.7%, and it was higher in non-AC than AC to campus.

**Fig. 1:**
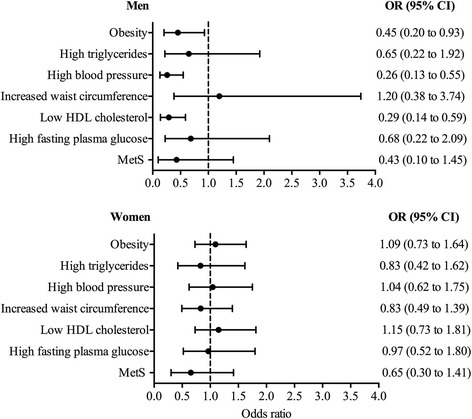
Multiple logistic regressions predicting unhealthy profile according to active walking to university categories (active or passive) using bivariate analysis by sex. Reference: Passive commuters (OR, 1.0). Analysis adjusted for age, body mass index (except for waist circumference included as dependent variable), physical activity, alcohol and tobacco intake, diet, and distance

The multiple logistic regressions for predicting an unhealthy profile showed that male walking commuters had a lower probability of having obesity [OR = 0.45 (CI 95% 0.25–0.93); *p* = 0.031], high blood pressure [OR = 0.26 (CI 95% 0.13–0.55); *p* < 0.001] and low HDL cholesterol [OR = 0.29 (CI 95% 0.14–0.59); *p* = 0.001] than did passive commuters (Fig. 1).

## Discussion

This study analyzes the relationship between AC to/from the university (walking), body composition, MetS and its components in a sample of Colombian university students. The percentage of active commuters (i.e., walkers to/from the university) was 65.3%; paradoxically, there were a greater percentage of PA levels (≥150 min per week) in passive compared to active commuters (28.3% vs 19.7%, respectively). Overall, male active commuters had a lower probability of having obesity, high blood pressure and low HDL cholesterol than did active commuters. Therefore, university students could benefit from participation in this behavior by increasing their knowledge regarding the importance and benefits of these sustainable modes of transportation [11]. However, the positive metabolic effects of active commuting reported based on the logistic regressions apply exclusively to male participants, which make only 21.4% of the study sample and therefore caution is warranted when interpreting data.

During transition from adolescence to young adulthood, the risk of physical inactivity and obesity becomes more prevalent; thus, it is essential to have a better understanding of the students’ behaviors to develop strategies to prevent certain of these negative health outcomes. With respect to prevalence of obesity, our results are consistent with previous studies, which have determined that daily walking to different places (e.g., to/from school or work) is not sufficient stimulus to generate changes in the body composition in women, although it does generate changes in males [14, 26]. In a young adult population, Gordon-Larsen et al. [14] suggested that the reason for the lack of associations for women could be that they may have a lower intensity of activity during AC. Since there were few university students who cycle to the university, we cannot confirm this hypothesis about AC intensity. In contrast, other studies have shown a relationship between AC and obesity in both sexes [27, 28]. Therefore, walking behavior can be integrated into other activities beyond leisure into AC and overall lifestyle activity, which could reduce obesity prevalence.

A recent meta-analysis from 16 prospective studies suggests a negative linear association between leisure-time physical activity and the incident MetS across the identified cohorts with a reduction of MetS risk by 8% per 10 MET h/week increments in leisure-time physical activity. With double the minimum recommended levels, the risk was reduced 20% [29]. In this respect, walking commuting has been recommended as a feasible method of incorporating greater levels of PA into daily life [30]. A previous meta-analysis reported that AC (no differentiation between commuting by walking and cycling) is related to a lower risk of adverse cardiovascular outcomes [30]. Another research study from the UK BIOBANK study consisting of 263,540 adults suggested that walking commuting is associated with a lower risk of cardiovascular disease incidence and mortality in a dose-dependent manner and independent of a range of confounding factors [11]. Regarding the Latino population, a recent study published by Steell et al. [31], based on data from participants aged ≥15 years from the 2009–10 Chilean National Health Survey, showed an association between AC and lower adiposity and odds for cardiovascular risk factors (diabetes and MetS) but did not identify any association between active commuting and HDL-cholesterol or blood pressure. In this sense, the results of these studies showed inconsistent conclusions about AC and metabolic health. Our study suggested that active commuters had a lower probability of having high blood pressure and low HDL cholesterol compared to walking commuters. Confirming our findings, the CARDIA Study reported that AC was inversely related with triglyceride levels, blood pressure, and insulin level in males [14]. In addition, two studies in a Chinese adult population published by Hu et al. [26, 32] observed no association between AC and diastolic blood pressure or HDL-cholesterol in males, but the association was significant for women. As previously described, the lack of information regarding the intensity of PA could explain the discrepancies observed between studies.

### Limitations

Due to the cross-sectional nature of the study design, we were unable to draw causal relationships. In addition, we analyzed relatively healthy university students; thus, the generalization of our results is limited to this population. The use of a self-reported method introduces further limitations, including the possibility of bias and unknown validity or reliability. Also, FUPRECOL Adults Study participants were asked to provide their main commuting mode; thus, mixed-mode journeys were not captured. It is therefore likely that the students who reported using a form of public transport as their main mode of transportation were highly heterogeneous in terms of the levels of PA that their commutes entailed. Finally, other pertinent factors such as sedentary behaviour and sleep were not considered.

## Conclusions

AC to and from the university could be an useful strategy to improve health in university students, since male walking commuters showed a lower probability of having obesity, low HDL-C and high blood pressure than did passive commuters. Taken together, these findings suggest that in young adulthood, a key life-stage for the development of obesity and MetS, AC could be a useful means of increasing daily PA levels, thereby promoting better cardiometabolic health. However, further research is warranted to understand the relationship between AC and cardiovascular risk factors in university students.

## Abbreviations

AC: Active commuting
BMI: Body mass index
HDL-C: High-density lipoprotein-cholesterol
LDL-C: Low-density lipoprotein-cholesterol
MetS: Metabolic syndrome
PA: Physical activity
TC: Total cholesterol
TG: Triglycerides
WC: Waist circumference

## References

[1] Zimmet P, Magliano D, Matsuzawa Y, Alberti G, Shaw J (2005). The metabolic syndrome: a global public health problem and a new definition. J Atheroscler Thromb.

[2] Spalding A, Kernan J, Lockette W (2009). The metabolic syndrome: a modern plague spread by modern technology. J Clin Hypertens.

[3] Haskell WL, Lee I-M, Pate RR, Powell KE, Blair SN, Franklin BA (2007). Physical activity and public health: updated recommendation for adults from the American College of Sports Medicine and the American Heart Association. Circulation.

[4] Booth FW, Roberts CK, Thyfault JP, Ruegsegger GN, Toedebusch RG (2017). Role of inactivity in chronic diseases. Evolutionary insight and pathophysiological mechanisms. Physiol Rev.

[5] Encuesta Nacional de Situación Nutricional. Encuesta Nacional de la Situación Nutricional en Colombia ENSIN-2015; https://www.minsalud.gov.co/salud/publica/epidemiologia/Paginas/encuesta-nacional-de-situacion-nutricional-ensin.aspx. Accessed 15 Mar 2018.

[6] Molina-García J, Queralt A, Castillo I, Sallis JF (2015). Changes in physical activity domains during the transition out of high school: psychosocial and environmental correlates. J Phys Act Health.

[7] Clemente FM, Nikolaidis PT, Martins FML, Mendes RS (2016). Physical activity patterns in university students: Do they follow the public health guidelines?. PLoS One.

[8] Flint E, Cummins S, Sacker A (2014). Associations between active commuting, body fat, and body mass index: population based, cross sectional study in the United Kingdom. BMJ.

[9] Gutiérrez-Zornoza M, Sánchez-López M, García-Hermoso A, González-García A, Chillón P, Martínez-Vizcaíno V (2015). Active commuting to school, weight status, and cardiometabolic risk in children from rural areas: the Cuenca study. Health Educ Behav.

[10] Ramírez-Vélez R, García-Hermoso A, Agostinis-Sobrinho C, Mota J, Santos R, Correa-Bautista JE (2017). Cycling to school and body composition, physical fitness, and metabolic syndrome in children and adolescents. J Pediatr.

[11] Celis-Morales CA, Lyall DM, Welsh P, Anderson J, Steell L, Guo Y (2017). Association between active commuting and incident cardiovascular disease, cancer, and mortality: prospective cohort study. BMJ.

[12] Bopp M, Behrens TK, Velecina R (2014). Associations of weight status, social factors, and active travel among college students. Am J Health Educ.

[13] Bopp M, Bopp C, Schuchert M (2015). Active transportation to and on campus is associated with objectively measured fitness outcomes among college students. J Phys Act Health.

[14] Gordon-Larsen P, Boone-Heinonen J, Sidney S, Sternfeld B, Jacobs DR, Lewis CE (2009). Active commuting and cardiovascular disease risk: the CARDIA study. Arch Intern Med.

[15] Ramírez-Vélez R, Correa-Bautista JE, Sanders-Tordecilla A, Ojeda-Pardo ML, Cobo-Mejía EA, Castellanos-Vega RP (2017). Percentage of body fat and fat mass index as a screening tool for metabolic syndrome prediction in Colombian university students. Nutrients.

[16] Marfell-Jones MJ, Stewart A, De Ridder J (2012). International standards for anthropometric assessment.

[17] Rodríguez-Rodríguez F, Cristi-Montero C, González-Ruíz K, Correa-Bautista JE, Ramírez-Vélez R (2016). Bioelectrical impedance vector analysis and muscular fitness in healthy men. Nutrients.

[18] Mancia G, Fagard R, Narkiewicz K, Redán J, Zanchetti A, Böhm M (2013). 2013 practice guidelines for the management of arterial hypertension of the European Society of Hypertension (ESH) and the European Society of Cardiology (ESC): ESH/ESC task force for the Management of Arterial Hypertension. J Hypertens.

[19] Friedewald WT, Levy RI, Fredrickson DS (1972). Estimation of the concentration of low-density lipoprotein cholesterol in plasma, without use of the preparative ultracentrifuge. Clin Chem.

[20] Alberti K, Eckel R, Grundy S, Zimmet P, Cleeman J, Donato K (2009). Harmonizing the metabolic syndrome. A joint interim statement of the IDF task force on epidemiology and prevention; NHL and Blood Institute; AHA; WHF; IAS; and IA for the study of obesity. Circulation.

[21] Chillón P, Herrador-Colmenero M, Migueles JH, Cabanas-Sánchez V, Fernández-Santos JR, Veiga ÓL (2017). Convergent validation of a questionnaire to assess the mode and frequency of commuting to and from school. Scand J Public Health.

[22] Herrador-Colmenero M, Pérez-García M, Ruiz JR, Chillón P (2014). Assessing modes and frequency of commuting to school in youngsters: a systematic review. Pediatr Exerc Sci.

[23] Molina-García J, Sallis JF, Castillo I (2014). Active commuting and sociodemographic factors among university students in Spain. J Phys Act Health.

[24] Ramírez-Vélez R, Agredo RA (2012). The Fantastic instrument’s validity and reliability for measuring Colombian adults’ life-style. Rev Salud Publica.

[25] Thanopoulou A, Karamanos B, Angelico F, Assaad-Khalil S, Djordjevic P, Katsilambros N (2006). Epidemiological evidence for the non-random clustering of the components of the metabolic syndrome: multicentre study of the Mediterranean Group for the study of diabetes. Eur J Clin Nutr.

[26] Hu G, Pekkarinen H, HÄnninen O, Yu Z, Guo Z, Tian H (2002). Commuting, leisure-time physical activity, and cardiovascular risk factors in China. Med Sci Sports Exerc.

[27] Lindström M (2008). Means of transportation to work and overweight and obesity: a population-based study in southern Sweden. Prev Med.

[28] Flint E, Cummins S (2016). Active commuting and obesity in mid-life: cross-sectional, observational evidence from UK Biobank. Lancet Diabetes Endocrinol.

[29] Zhang D, Liu X, Liu Y, Sun X, Wang B, Ren Y (2017). Leisure-time physical activity and incident metabolic syndrome: a systematic review and dose-response meta-analysis of cohort studies. Metabolism.

[30] Hamer M, Chida Y (2008). Active commuting and cardiovascular risk: a meta-analytic review. Prev Med.

[31] Steell L, Garrido-Méndez A, Petermann F, Díaz-Martínez X, Martínez MA, Leiva AM, et al. Active commuting is associated with a lower risk of obesity, diabetes and metabolic syndrome in Chilean adults. J Public Health. 2017:1–9. 10.1093/pubmed/fdx092 28977515

[32] Hu G, Pekkarinen H, Hänninen O, Tian H, Guo Z (2001). Relation between commuting, leisure time physical activity and serum lipids in a Chinese urban population. Ann Hum Biol.

